# Molecular basis of transport of surface functionalised gold nanoparticles to pulmonary surfactant†

**DOI:** 10.1039/d2ra01892f

**Published:** 2022-06-17

**Authors:** Fengxuan Jiao, Sheikh I. Hossain, Jianbing Sang, Suvash C. Saha, YuanTong Gu, Zak E. Hughes, Neha S. Gandhi

**Affiliations:** School of Mechanical Engineering, Hebei University of Technology Tianjin 300401 PR China; School of Life Science, University of Technology Sydney 81 Broadway Ultimo NSW 2007 Australia; School of Mechanical and Mechatronic Engineering, University of Technology Sydney 81 Broadway Ultimo NSW 2007 Australia; School of Mechanical Medical & Process Engineering, Queensland University of Technology 2 George Street, GPO Box 2434 Brisbane QLD 4000 Australia; School of Chemistry and Biosciences, The University of Bradford Bradford BD7 1DP UK Z.Hughes@bradford.ac.uk; Centre for Genomics and Personalised Health, School of Chemistry and Physics, Queensland University of Technology 2 George Street, GPO Box 2434 Brisbane QLD 4000 Australia neha.gandhi@qut.edu.au

## Abstract

Ligands like alkanethiol (*e.g.* dodecanethiol, hexadecanethiol, *etc.*) and polymers (*e.g.* poly(vinyl pyrrolidone), polyethylene glycol-thiol) capped to the gold nanoparticles (AuNPs) are widely used in biomedical field as drug carriers and as promising materials for probing and manipulating cellular processes. Ligand functionalised AuNPs are known to interact with the pulmonary surfactant (PS) monolayer once reaching the alveolar region. Therefore, it is crucial to understand the interaction between AuNPs and PS monolayers. Using coarse-grained molecular dynamics simulations, the effect of ligand density, and ligand length have been studied for two classes of ligands on a PS model monolayer consisting of DPPC, POPG, cholesterol and SP-B (mini-peptide). The ligands considered in this study are alkanethiol and polyethylene glycol (PEG) thiol as examples of hydrophobic and hydrophilic ligands, respectively. It was observed that the interaction between AuNPs and PS changes the biophysical properties of PS monolayer in compressed and expanded states. The AuNPs with hydrophilic ligand, can penetrate through the monolayer more easily, while the AuNPs with hydrophobic ligand are embedded in the monolayer and participated in deforming the monolayer structure particularly the monolayer in the compressed state. The bare AuNPs hinder to lower the monolayer surface tension value at the interface, however introducing ligand to the bare AuNPs or increasing the ligand length and density have an impact of lowering of monolayer surface tension to a minor extent. The simulation results guide the design of ligand protected NPs as drug carriers and can identify the nanoparticles' potential side effects on lung surfactant.

## Abstract 2

Molecular-level observations of the behavior of ligand functionalised gold nanoparticles with a lipid monolayers.

## Graphical abstract

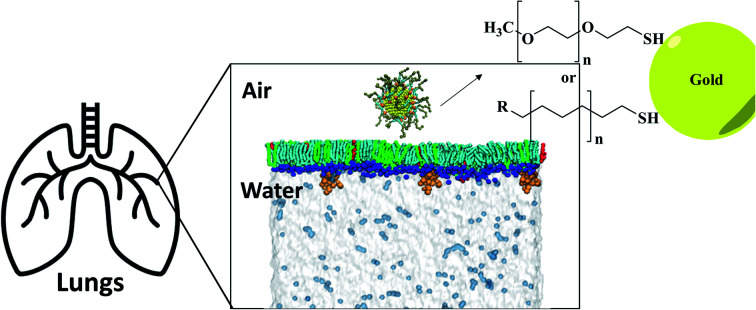

## Introduction

Metallic nanomaterials such as gold (AuNPs) have attracted considerable attention from researchers for their applications in drug delivery, bio-imaging, specific targeting and bio-sensing.^1–6^ AuNPs are easy to prepare, have good stability in biological media and can be subjected to wide surface modification.^7,8^ AuNPs exert two-sided effects.^9^ On the one hand, they have been used for respiratory applications that aim to overcome conventional drugs' limitations. On the other hand, they could induce pulmonary diseases and even injuries in other tissues. Once inhaled, AuNPs need to cross pulmonary surfactant (PS) of lung alveoli before being delivered into the lung airspaces.^10^ PS monolayers are composed of ∼90% lipids (phospholipids and cholesterol) and ∼10% proteins. PS forms as the breathing gas–liquid interface of lung alveoli, where it reduces the surface tension, prevents alveolar collapse, and has the function of host defense. Researchers have investigated the properties of the PS to deliver drugs on the alveolar interface. As mentioned above, nanoparticles can quickly enter the respiratory system, interact with PS monolayer, and affect the lung's normal function, leading to lung disease. A key issue is balancing the side effects of NPs and the penetrating ability of NPs.^9^ Therefore, it is important to evaluate the interaction between AuNPs and PS monolayer if researchers use AuNPs as drug carriers. There are many convincing experimental pieces of evidence indicating that interaction between inhaled nanoparticles and PS monolayer will affect biophysical functions of the PS.^11^

Naked AuNPs easily aggregate due to the high ionic strength of many biological fluids and the non-specific interaction of nanoparticles with biomolecules, such as proteins or DNA. Therefore, researchers made significant attempts to optimise nanoparticles' shape, size, and density to improve drug delivery efficiency and prevent aggregation.^10–13^ One of the most common methods of tuning the properties of AuNPs is *via* the use of ligands adsorbed or bound to the surface of the metallic gold core. The ligands form an outer organic monolayer on the nanoparticle and are not as influential in determining the physical properties as the core. They are fundamentally important in modifying the interactions of the nanoparticle with its environment. For decades, oligonucleotide, drugs, peptides and polymers have been covalently conjugated to the spherical AuNPs through the thiol group.^14–16^ Poly(ethylene glycol)-thiol (PEG-SH) and alkanethiol are commonly used to functionalise the surface of AuNPs. Alkanethiol-functionalised AuNPs possess higher stability when compared to most other AuNPs due to the thiol–gold interactions and van der Waals attractions between the neighbouring ligands.^17^ Molecular dynamics (MD) simulations of alkanethiol functionalised AuNPs have shown a negative effect on lipid bilayers wherein the alkane ligands reached out to lipid tails in the inner region, pushing the membrane's headgroups apart transport process. The extent of penetration of alkanethiol functionalised AuNPs was dependent on the size, surface coverage and ligand length.^18,19^ The effect of concentrations in a range of 0.1 to 0.5 mol% of hydrophobic alkylated (*e.g.* hexadecanethiolate) AuNPs with an average 1.5–3 nm diameter on the biophysical properties of lung surfactant monolayers of dipalmitoylphosphatidylcholine (DPPC) has also been studied using experimental techniques. Techniques like atomic force microscopy, Brewster angle microscopy, surface pressure–area (π–A) and ellipsometric isotherms suggested that hydrophobic NPs induce PS inhibition much more readily.^20,21^ The results also demonstrated that alkanethiolate-capped AuNPs formed random aggregates in monolayers of phospholipids that exhibit a liquid-expanded phase during lateral film compression.

PEG-SH is a prevalent coating strategy to lengthen the circulation time of biomedicines in the bloodstream and minimise the aggregation in blood.^22–24^ PEG-SH can form a corona on the surface of the NPs, reduce specific protein adsorption thus reduce side effect on PS monolayer, however, excessive PEG coronas may reduce translocation efficiency.^25^ Bouzas investigated the impact of PEG-SH functionalised NPs on freshly purified primary cultures of rat alveolar type II (ATII) cells and found that PEG-SH functionalised NPs reduce the possible negative effects on the metabolism of PS carried out by ATII cells.^26^ Another experimental study found that systemic uptake of mice airway is higher for PEG-SH functionalised AuNPs compared to citrated nanoparticles.^27^ Several MD simulations have been reported to study the conformation of PEG-SH AuNP in solution and on the membranes. Oroskar *et al.* found that gold nanoparticles protected by PEG-SH ligands may lead to increased cell cytotoxicity, but have a less side effect on the integrity of lipid bilayer due to favourable interactions with the lipid headgroups.^28^ AuNPs wherein the alkyl thiol ligand terminals have ammonium or carboxylate groups can spontaneously adhere to the bilayer surface or penetrate the bilayer.^29^ This penetration is mainly governed by the electrostatic interactions between the functionalised ligand terminals of the AuNPs and the bilayer head groups. Another similar MD study of lipid monolayers and PEG-SH functionalised AuNPs (bare gold NP with a radius of about 3.2 nm and PEG chains with charged termini) suggested similar interactions.^30^ The positively charged NPs will adhere to the lipid heads, and the negatively charged or neutral NPs can possibly deplete the surfactant proteins or lipids through penetration. All these studies showed potential increase in the surface tension of PS monolayers. MD simulations of DPPC monolayer and rigid spherical NP (PEG-SH chain length = 9, diameter = 3 and 5 nm) at the air water interface suggested that PEG-SH functionalised NPs can readily penetrate the aqueous phase with little or no disturbance on the DPPC monolayer.^31^

MD simulations are an effective way to research the translocation of NPs into monolayer at a molecular level that is otherwise not available using experimental biophysical characterisation techniques. Coarse-grained molecular dynamics (CGMD) simulations have been used to research the structural changes in PS due to the inhalation of different NPs like carbon fullerene, nanotubes, graphene, silica, calcium sulfate *etc.*,^31–40^ drug molecules, peptides and polymers.^30,41–45^ In this work, we used CGMD simulations to research the effects of AuNPs with different ligand density, ligand length, and hydrophobicity on PS monolayers at two different area per lipids (APLs). The hydrophobicity of the NP is controlled by using PEG (hydrophilic) and alkanethiol (hydrophobic) ligands. APL and PS phase are regulated by surface tension of monolayer. The AuNPs with varying coatings of ligand interact with the PS monolayers and therefore can affect the efficacy of nanoparticle-based systems for the purpose of delivery of drugs.

## Experimental/computational methods

Coarse-grained (CG) models allow simulation of systems to be performed at greater length- and time-scales compared to with all atom force field (FF) simulations.^46,47^ The CG model used in study is based on the Martini FF which has been widely used to simulate biomolecules. The MARTINI model was extended and used to model inorganic systems like silicates and modified montmorillonite.^48,49^ The Martini FF uses a four-to-one mapping, *i.e.* on average four heavy atoms and associated hydrogens are represented by a single interaction centre.

**Fig. 1:**
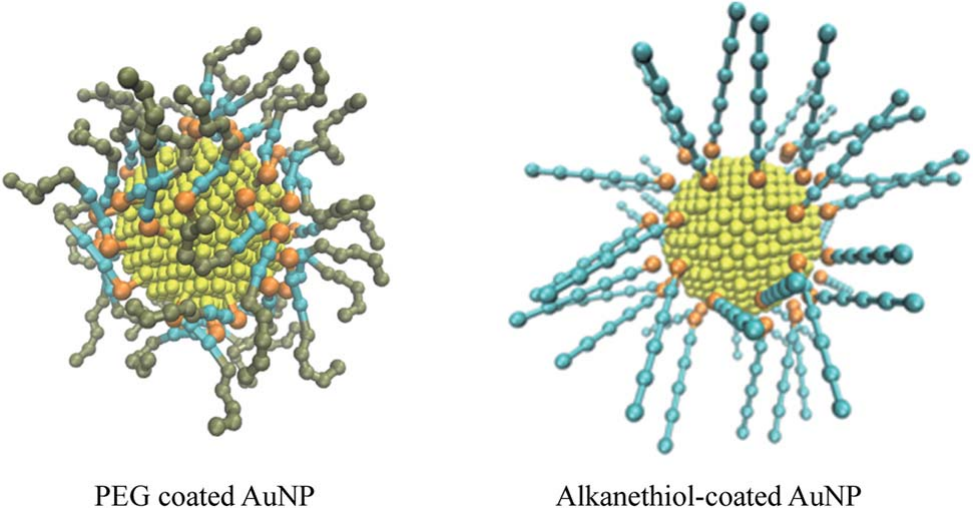
Structure of coarse-grained PEG-SH functionalised and alkanethiol-functionalised AuNP. The gold, sulfur, alkyl chain and ethyleneoxide sites are coloured yellow, orange, cyan and tan, respectively. The snapshots are of systems with a coating density of 1.56 chain per nm^2^, with a chain length of 2 and 5 for the PEG and alkanethiol systems respectively.

The Martini force parameters for DPPC, POPG, cholesterol and water were taken from the Martini website. The CG model in MARTINI for AuNPs were prepared as outlined in previous studies by Hossain *et al.*^32^ The AuNP core was made with OpenMD NP builder using Sutton-Chen force field.^50^ The MARTINI CG model for alkanethiol (Table 1) and PEG-SH (Table 2) were adopted from the work of Lin *et al.*^51^ – The atomistic-to-CG mapping of atoms in PEG monomer is 3 : 1, in alkane is 4 : 1. Au and S atoms are mapped on a 1 : 1 level. The Au atoms were mapped to a C5 bead, the alkane was mapped to C1 bead, the S atoms were mapped to an N0 bead, the PEG monomer was mapped to an SN0 bead, and its terminal monomer was mapped to an SN2 bead in the CG force field. The PEG ligand and thiol ligand are assembled on the surface of gold nanoparticles with PACKMOL package.^52^ AuNPs models are shown in Fig. 1.

**Table 1:** Force field parameters of the CG AuNP and alkanethiol

Bead name	Bead type	Bonds^a^			Angles^b^			Constraints	
		Beads	*b*_0_	*K*_b_	Bead type	*θ*_0_	*K*_θ_	Beads	Type
Au	C5	S–(CH_2_)_4_	0.445	1250	S–(CH_2_)_4_–(CH_2_)_4_	180	25	Au–Au	Fix rigid
S	N0	(CH_2_)_4_–(CH_2_)_4_	0.470	1250	(CH_2_)_4_–(CH_2_)_4_–(CH_2_)_4_	180	25	Au–S	Fix rigid
(CH_2_)_4_	C1								

a *b*_0_ in nm, *K*_b_ in kJ mol^−1^ nm^−2^.

b *θ*_0_ in deg., *K*_θ_ in kJ mol^−1^ rad^−2^.

**Table 2:** Coarse-grained force field parameters for PEG-SH

Building block	Non-bonded (LJ)^a^		Bonded						
			Bond^b^		Angle^c^		Dihedral^d^		
	*σ*	*ε*	*b*_0_	*K*_b_	*θ*_0_	*k*_θ_	*ϕ*_0_	*K* ^*ϕ*^	*n*
PEG monomer	0.43	3.375	0.33	17 000	130	85	180	1.96	1
							0	0.18	2
							0	0.33	3
							0	0.12	4

a *σ* in nm, *ε* in kJ mol^−1^ nm^−2^.

b *b*_0_ in nm, *K*_b_ in kJ mol^−1^ nm^−2^.

c *θ*_0_ in deg., *K*_θ_ in kJ mol^−1^ rad^−2^.

d *ϕ*_0_ in deg., *K*^*ϕ*^ in kJ mol^−1^.

**Fig. 2:**
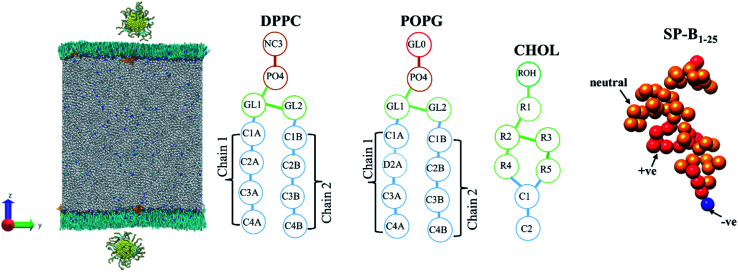
The initial coarse-grained structure of model PS monolayer and ligand functionalised AuNP at the vacuum–water interface, and schematics of the molecules that make up the PS monolayer.

The monolayer systems each consisting of 1035 lipids were built from splitting a bilayer with an INSANE (python script)^53^ and the molar ratio of DPPC, POPG, cholesterol and SP-B_1–25_ is approximately 70 : 30 : 10 : 1, matching the make-up of biological systems. The two lung surfactant monolayers are packed in a periodic boundary box of ∼25 × ∼25 × ∼60 nm. The lipid plane is parallel to the *XY* plane of the system, a ∼21 nm water slab with about 100 000 CG water beads and with a density of 1 kg dm^−3^ is placed between the two monolayers. NaCl with ionic concentration of 150 mM was added to the water slab, with an additional number of water beads are replaced by Na^+^ beads to ensure the charge neutrality of the system. The system was minimized using the steepest descent algorithm and equilibrated for 500 ns in the NPT ensemble using Berendsen pressure barostat^54^ at surface tensions of 0 and 23 mN m^−1^ to obtain compressed and expended state monolayers, respectively. The compressibility was set to 4.5 × 10^−5^ bar^−1^ along *XY* plane and 0 bar^−1^ along *Z*-axis. This simulation protocol is similar to the one described in our published work.^32,33^ The nanoparticles are initially placed in the vacuum close (within ∼1 nm) to the equilibrated monolayers, as shown in Fig. 2.

The CGMD simulations were performed using GROMACS 2019. Simulations were performed for two different phases of the PS monolayers with different area per lipids (APL), the compressed (liquid condensed (LC) phase, APL = 0.48 nm^2^) and the expanded (liquid-condensed and liquid-expanded coexistence phase (LC + LE), APL = 0.55 nm^2^). The gold core was 3 nm in diameter whereas both the chain length and the chain density were varied for both ligands. Table 3 outlines a full list of the systems simulated. Specifically, we modelled AuNPs with PEG grafting densities ranging from 1.56–3.89 chain per nm^2^ and chain length with 1–4 monomers with fixed density of 2.33 chain per nm^2^. The alkanethiol–AuNP densities were in range from 0.78–3.11 chain per nm^2^ and chain length with 2–5 monomers with fixed density of 2.33 chain per nm^2^. Chain density is defined by the number of alkanethiol linkers bonded to the NP per surface area. The chain length is defined as the number of C1 type beads (see Table 1) in the chains.

**Table 3:** Summary of the systems simulated, the equilibrated surface tension (nN m^−1^) and the behaviour of the nanoparticle

Ligand			Compressed (APL = 0.48 nm^2^)		Expanded (APL = 0.54 nm^2^)	
Type	Chain density/chains per nm^2^	Chain length/sites	Surface tension/mN m^−1^	System behaviour	Surface tension/mN m^−1^	System behaviour
PEG-SH	1.56	2	17.1 ± 0.8	Enters water layer, PS starts to form a fold	27.4 ± 2.5	Enters water layer
	2.33	2	13.7 ± 1.8		29.5 ± 1.2	
	3.11	2	11.9 ± 0.9		31.7 ± 2.0	
	3.89	2	9.5 ± 1.1		32.8 ± 1.2	
	2.33	1	16.8 ± 0.5	Enters water layer, PS starts to form a fold	27.7 ± 2.5	Enters water layer
	2.33	2	13.7 ± 1.8		29.5 ± 1.2	
	2.33	3	17.1 ± 0.6		27.4 ± 1.0	
	2.33	4	23.2 ± 2.2		25.9 ± 2.2	
Alkanethiol	0.78	2	18.0 ± 0.2	Embeds in PS, fold starts to form	20.1 ± 0.9	Embeds in PS
	1.56	2	15.1 ± 0.3		18.8 ± 0.6	
	2.33	2	14.4 ± 0.4		21.6 ± 0.6	
	3.11	2	14.3 ± 0.1		27.1 ± 0.2	
	1.56	2	15.1 ± 0.3	Embeds in PS, fold starts to form	19.0 ± 0.6	Embeds in PS
	1.56	3	14.5 ± 0.5		18.8 ± 0.6	
	1.56	4	14.7 ± 0.6		24.2 ± 0.2	
	1.56	5	14.6 ± 0.3		25.5 ± 0.9	

The cutoff of van der Waals interactions was 1.2 nm, the LJ potential shifted to zero between 0.9 nm and 1.2 nm and the coulombic potential with a cutoff of 1.2 nm smoothly shifted to zero from 0 to 1.2 nm. In the presence of functionalised AuNPs, each system was minimized again. MD simulations were performed in the NVT ensemble the lipids (including cholesterol), proteins, water and ions, and AuNPs independently coupled to velocity rescale thermostats at 310 K. In all MD simulations, the equations of motion were integrated *via* the leapfrog algorithm using a 15 fs time step.

Each system was simulated for 1050 ns at least twice. To confirm that 1050 ns was a suitable timescale to ensure equilibration some systems were simulated for 3000 ns. Representative data is shown for systems with a chain density of 2.33 chain per nm^2^ and a chain length of 2 (data shown in ESI†). The results of the extended simulations show that systems had equilibrated by <500 ns with respect to the properties of interest in the current work. Unless mentioned otherwise analysis was done over the last 300 ns of the simulation and averaged over all the independent runs.

The surface tensions of each monolayer are calculated by following formula:
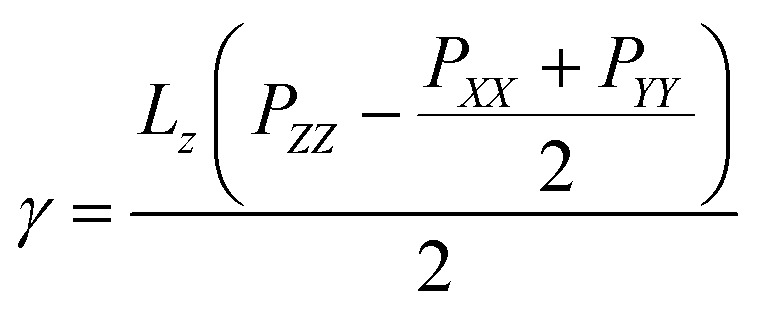
where *γ* is the monolayer surface tension, *L*_*z*_ is the box length in the normal direction, *P*_*ZZ*_ is the pressure along the normal of the monolayer, 
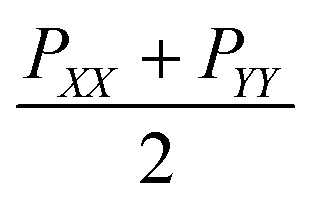
 is the lateral pressure.

## Results and discussion

**Fig. 3:**
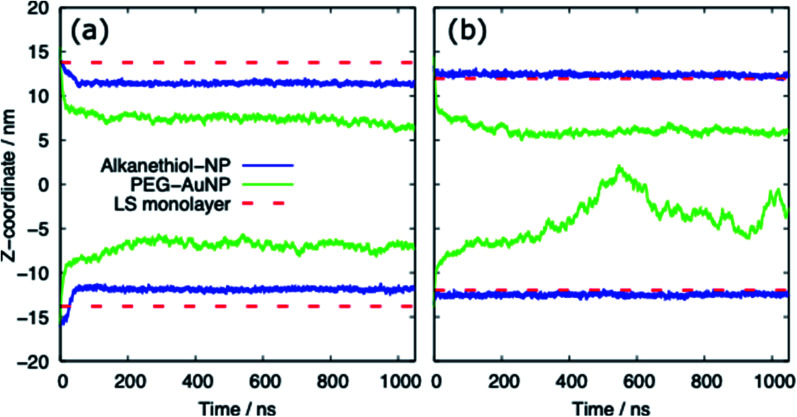
The *z*-coordinate of the centre of mass of the AuNP as a function of simulation time for (a) the compressed (APL = 0.48 nm^2^) and (b) expanded (APL = 0.54 nm^2^) phases. The centre of mass of the lipids in each monolayer is shown by the dashed red lines. Representative data shown for systems with a chain density of 2.33 chain per nm^2^ and a chain length of 2.

Table 3 provides a summary of the systems simulated, with the surface tension and behaviour of the nanoparticle and PS monolayer. There is a clear distinction between the behaviour of the alkanethiol functionalised NPs and the PEG functionalised NPs. Fig. 3 shows the *z*-coordinate of the centre of mass of the AuNPs as a function of simulation time. By ∼50 ns the AuNPs have diffused to/through the PS monolayer. The different hydrophobicities of the two NP shells leads to very different behaviour.

### Interaction of PS monolayers with alkanethiol-functionalised AuNPs

**Fig. 4:**
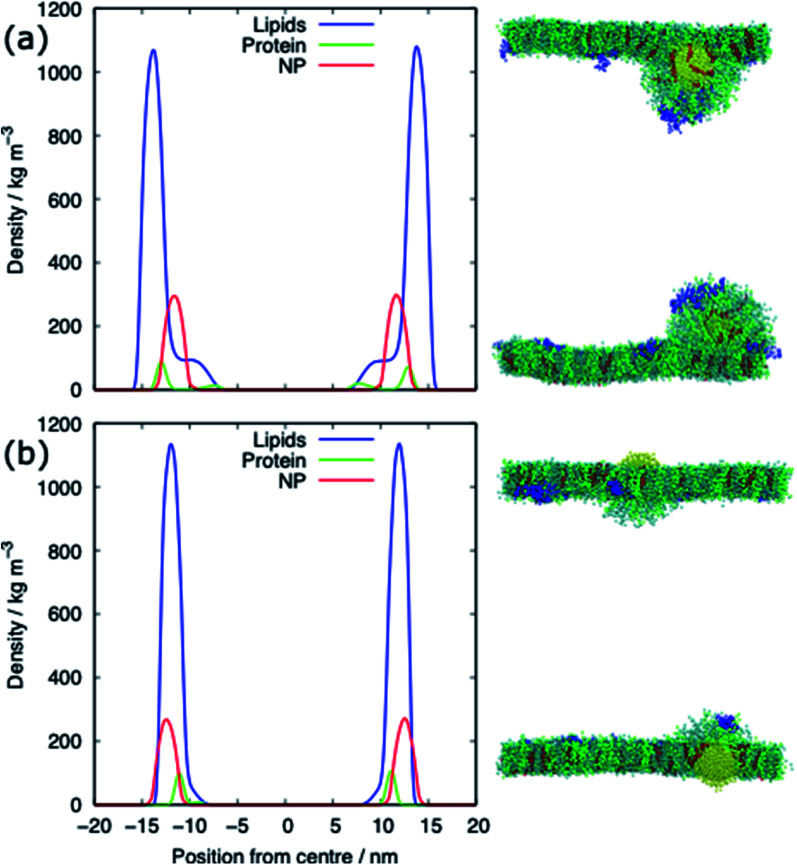
Density profiles and representative snapshots of the alkyl-functionalised AuNP interacting with (a) the compressed (APL = 0.48 nm^2^) and (b) the expanded (APL = 0.54 nm^2^) phases. Data shown for the alkanethiol–AuNP with a chain density of 2.33 chain per nm^2^ and a chain length of 2.

The alkanethiol-functionalised AuNPs have both a hydrophobic core and a hydrophobic shell and so become embedded within the PS monolayer. Fig. 4 shows density profiles and representative snapshots for the systems with an alkyl chain density of 2.33 chain per nm^2^ and a chain length of 2. The alkanethiol-functionalised AuNPs do not enter the water layer, the similar hydrophobicity of the ligands and lipid tails^34,37^ encourages the shell of the NP to interact with the lipids in the monolayer. For the compressed system (APL = 0.48 nm^2^) the embedding of the AuNP in the monolayer encourages a folding/buckling of the monolayer and the formation of a semi-vesicle of AuNP, lipids and proteins (Fig. 4(a)). In the expanded system the alkanethiol functionalised AuNP embedded within the monolayer but there is no evidence of buckling or formation of any semi-vesicles.

**Fig. 5:**
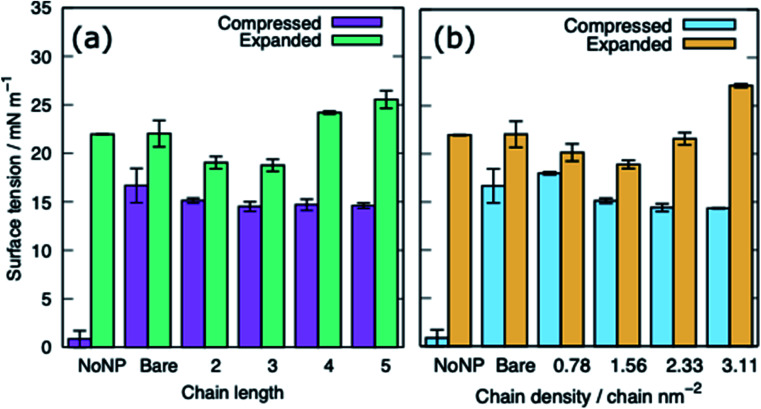
The surface tension of PS monolayers in the presence of alkanethiol-functionalised AuNPs, data given for systems with APLs 0.48 and 0.54 nm^2^, (a) effect of varying ligand chain length (density = 2.33 chains per nm^2^), (b) effect of varying ligand chain density (length = 2). Bare refers to a system with bare AuNP.

Fig. 5 shows the equilibrated surface tension of the PS monolayers when interacting with the various alkanethiol–AuNPs. For the compressed system the presence of an AuNP, bare or alkanethiol-functionalised, results in a large increase in the surface tension, from ∼0 to ∼15 mN m^−1^. This is due to the AuNP embedding in the PS and inducing some folding/buckling of the monolayer resulting in an increase in the surface tension compared to the control system. At a chain density of 2.33 chains per nm^2^, the surface tension for compressed system is largely insensitive to the chain length of the ligands (Fig. 5(a)), or even their presence (*i.e.* a chain length of 0). Neither is a strong effect on the surface tension observed for increasing the density of the chains (Fig. 5(b)). As such for compressed system we can conclude that the presence of the NP and the resultant promotion of monolayer buckling is the crucial factor, rather than the nature of the shell.

For the expanded system the presence of a bare AuNP causes a small increase in the surface tension, from ∼22 to ∼25 mN m^−1^ as the AuNP embeds in the PS and lipids and proteins are adsorbed to the NP. In contrast the interaction of an alkanethiol–AuNPs with a thin or patchy shell (*i.e.* low chain length or density) slightly lowers the surface tension compared to a system where no AuNP is present. It is only for AuNPs which have a high chain length and/or chain density that an increased surface tension (approximately comparable to that obtained in the present of the bare AuNP) is observed. The reason for this behaviour is that at low chain lengths/density the NP only moderately perturbs the PS monolayer, while occupying space in the monolayer, effectively reducing the APL. As the shell becomes thicker/denser the components of the PS monolayer interact with the NP more strongly, perturbing the monolayer and the surface tension increases.

### Interaction of PS monolayers with PEG-SH functionalised AuNPs

**Fig. 6:**
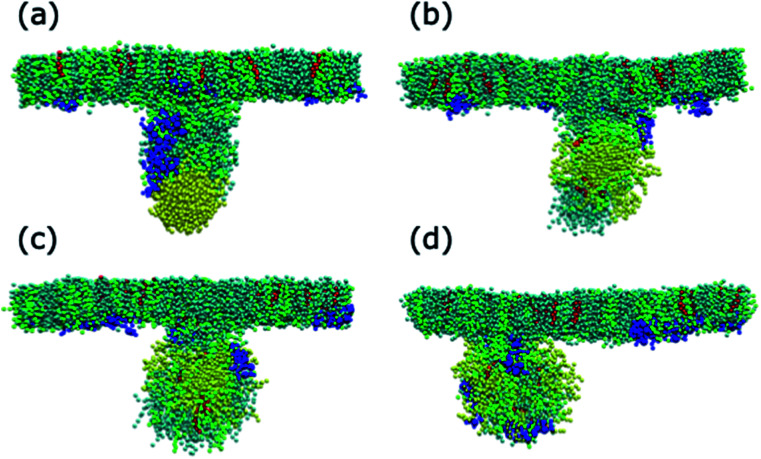
Representative snapshots of the equilibrated systems with PS monolayers in the compressed system, the PEG–AuNPs have a chain density of 1.56 chain per nm^2^ and a chain length of (a) 1, (b) 2, (c) 3 and (d) 4. DPPC, POPG, cholesterol, SP-B_1–25_ and the PEG–AuNP are coloured cyan, green, red, blue and yellow, respectively.

**Fig. 7:**
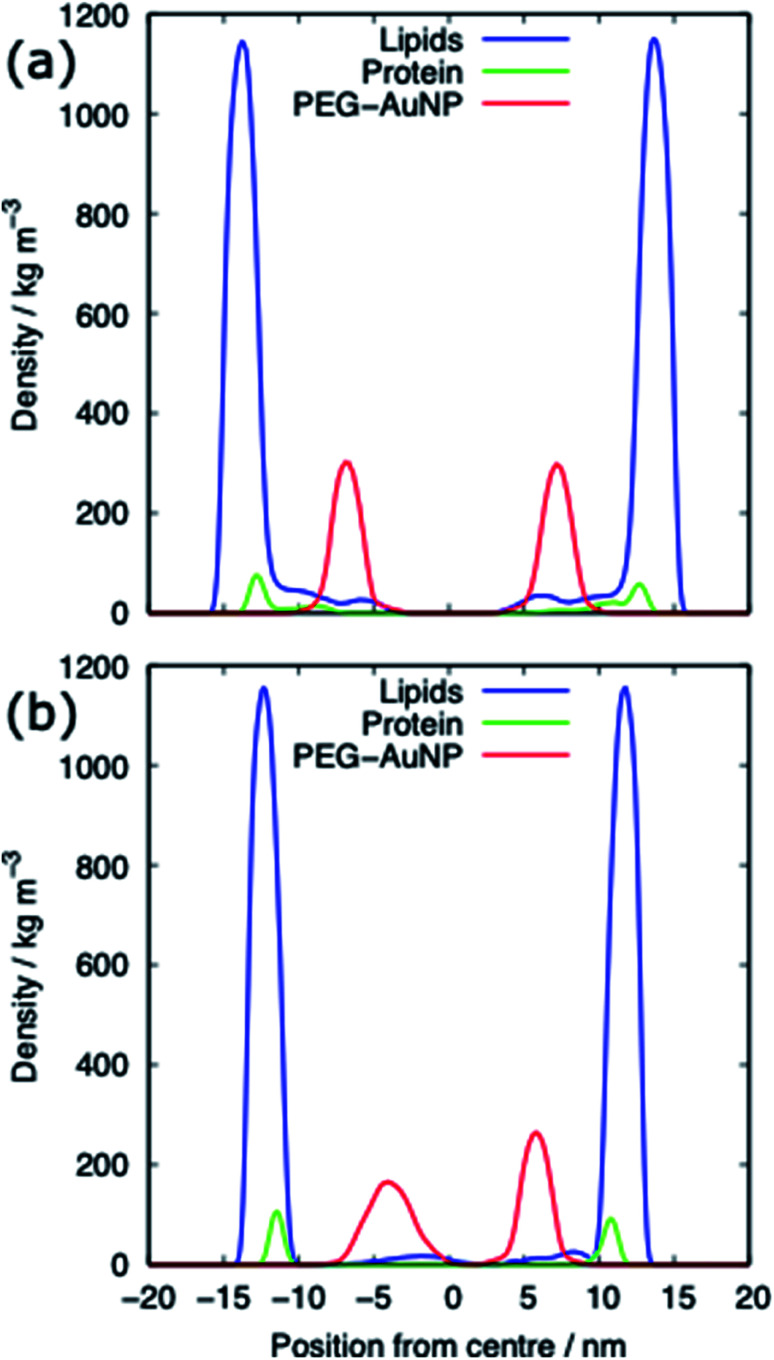
Density profiles of the PEG-SH functionalised AuNP PS monolayer systems for (a) the compressed (APL = 0.48 nm^2^) and (b) the expanded (APL = 0.54 nm^2^) phase. Data shown for the PEG–AuNP with a chain density of 2.33 chain per nm^2^ and a chain length of 2.

For both systems, compressed and expanded, the PEG-SH functionalised AuNP diffuses through the monolayer and enters the water layer. Representative snapshots illustrating the equilibrated systems are shown in Fig. 6 and density profiles of representative systems in Fig. 7. During the translocation process, some peptides and lipids typically become adsorbed to the PEG-SH functionalised AuNPs and are removed from the monolayers. Once the PEG-SH functionalised AuNPs has diffused through the PS the monolayer reforms and is not significantly perturbed. In the compressed system the PEG–AuNPs typically remain relatively close (within ∼10 nm) of the monolayer, while in the expanded system the nanoparticles may diffuse more deeply into the water layer (Fig. 3 and 7).

**Fig. 8:**
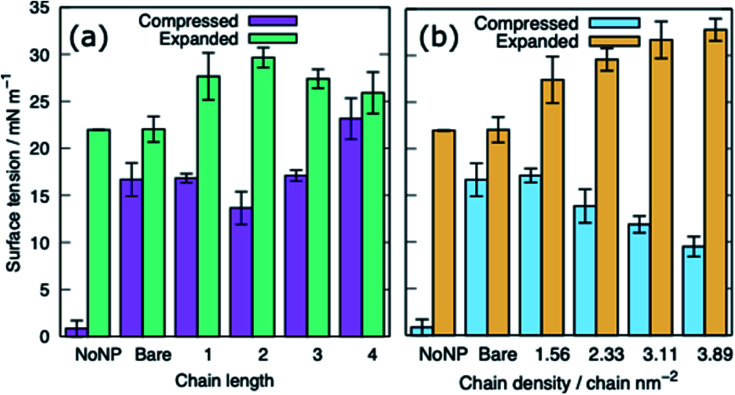
The surface tension of PS monolayers in the presence of PEG-SH functionalised AuNPs for the compressed and expanded systems: (a) varying ligand chain length (density = 1.56 chains per nm^2^), (b) varying ligand chain density (length = 2). Bare refers to a system with a bare AuNP.

The surface tension of PS monolayers interacting with PEG–AuNPs of different chain lengths/densities are given in Table 3 and Fig. 8. There is considerably more variance in the surface tension measurements than for the alkanethiol–AuNPs. In the expanded system the presence of the PEG–AuNPs causes an increase in surface tension above both the control (no NP present) and bare AuNP systems. This is due to the PEG–AuNP adsorbing lipids from the monolayers before diffusing into the water layer, so in effect increasing the APL of the system. As the chain density increases the PEG–AuNP becomes increasingly hydrophilic and is able to enter the water layer with increasing ease. The increase in surface tension is therefore linked to the ability of the PEG–AuNP to both adsorb PS monolayer material and to diffuse into the water layer.

In the compressed system the presence of the PEG–AuNP increases the surface tension of the systems compared to the control system as the presence of the NP perturbs the monolayer. However, the increase in surface tension becomes smaller as the chain density of the PEG–AuNP increases, as the more hydrophilic the NP the less the monolayer is disturbed (at the same time as the PEG–AuNPs remain relatively close to the PS monolayers they do not significantly affect the water layer). For chain lengths of 0 to 3 the increase in surface tension is approximately constant, however, for the longest chain length of 4 a higher surface tension is observed (in fact this is the highest surface tension observed for any compressed system simulated). The length of the chain for this PEG–AuNP give it a particular structure with a hydrophobic core, a hydrophobic inner shell and a hydrophilic outer shell. Thus, a PEG–AuNP with a chain density of 1.56 chain per nm^2^ and a chain length of 4 is able to strongly adsorb a significant amount of PS monolayer material but still able to diffuse into the water layer (Fig. 6(d)).

**Fig. 9:**
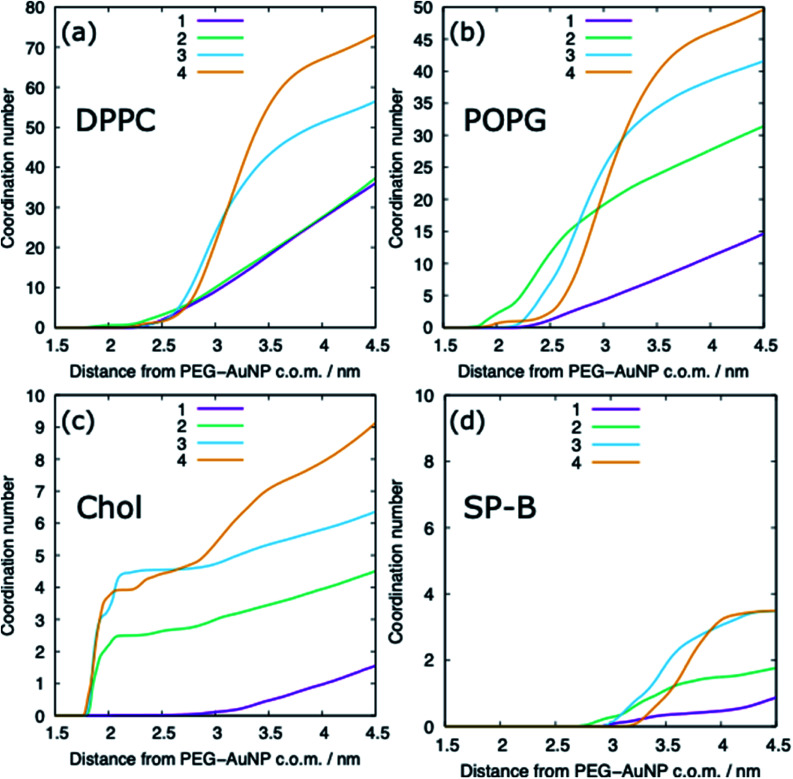
The molecular coordination number for (a) DPPC, (b) POPG, (c) cholesterol and (d) SP-B with the PEG–AuNP for different chain lengths, distances are between the centre of mass of the two molecules. Representative data is shown for the compressed system and for a PEG–AuNP with a chain density of 1.56 chain per nm^2^.

The adsorption of lipids (and proteins) to the PEG–AuNP varies with the chain length of the PEG ligands (Fig. 6 and 9). A previous study has shown that hydrophobic part of phospholipids interacts strongly with the hydrophobic bare AuNP, which supports the preference of cholesterol adsorption to the AuNP compared to DPPC and POPG.^36^ However, the presence of a ligand shell, and the properties of a shell, results in different adsorption behaviour. For the shortest PEG ligands, the chains lie very close to the core and there is limited adsorption of lipids, with POPG adsorbing preferentially to DPPC and cholesterol. As the chain length increases the amount of lipids that are able to adsorb to the PEG–AuNP increases and a genuine corona of lipids is present around the NP.

## Conclusion

In this paper, we have investigated the transport of ligand functionalised AuNPs on PS monolayer using coarse-grained molecular dynamics simulations. The PS monolayer was modelled using DPPC, POPG, cholesterol and SP-B_1–25_ is molar ratio of approximately 70 : 30 : 10 : 1. These PS were equilibrated at surface tensions of 0 and 23 mN m^−1^ to obtain compressed and expended states, representing alveolar tissue dynamics during breathing. We compared the different permeation behaviours for AuNPs (gold core with 3 nm in diameter) without surface ligands and functionalised nanoparticles with different ligand lengths and density and studied how the surface ligands change the details of the permeation process. The AuNPs diffused to/through the PS monolayer within ∼50 ns.

It was found that PEG-SH functionalised AuNP can penetrate through the monolayer to the water interface relatively easily compared to the monolayer embedded alkanethiol-functionalised AuNP. Hydrophobicity of the bare and alkanethiol-functionalised AuNP are the contributing factors for the AuNP to remain embedded in the PS monolayer, resulting in monolayer buckling and increasing the surface tension of the monolayer in the compressed state. A considerable variance in surface tension values of monolayers was noticed in the presence of the PEG-SH functionalised AuNP during expansion and compression states compared to monolayer systems without nanoparticle. However, the ligand density and length of PEG-SH AuNPs have moderate impact on the density profiles of PS components indicating that PEGylation of the metallic nanoparticles could omit unwanted interactions with the biophysical function of PS. As the ligand chain length is increased larger amounts of lipids and proteins adsorb to the PEG-SH AuNP suggesting that maximising the hydrophilicity of the ligand shell with short chain lengths and high chain density are a pathway to AuNPs that limit the disruption of the PS monolayers. Overall, our study provides a molecular level insight into the use of ligand functionalised AuNP which can be used to design novel pulmonary drug delivery systems.

Our coarse-grained simulations results provide a scope where one engineer the surface of AuNPs to create various biophysical changes in the lung surfactant monolayers for delivering the targeted drug/polymers/proteins.

Our model monolayers contain the main components of lung surfactant (saturated phospholipids, unsaturated phospholipids, cholesterol and a mini surfactant peptide) but there is some scope for improvements *e.g.* considering simulations to include triglycerides, oligomeric states of surfactant proteins and additional surface tensions of 5, 10 and 30 mN m^−1^. This study does not allow us to tell whether the concentration of the nanoparticle, aggregation or the charge of the functionalised ligand is a requirement to reduce monolayer stability. In near future, this study can be extended to include a certain concentration and charge of ligand functionalised AuNPs and gain a better understanding of the mechanisms for cytotoxicity or rupture of the monolayers.

## Data availability

The files with initial configurations, potential parameters and simulation parameters are stored in the Figshare repository (https://figshare.com/articles/dataset/Simulations_of_alkanethiol_AuNPs/19932455 and https://figshare.com/articles/dataset/Simulations_of_PEG-SH_AuNPs/19932293) to reproduce the MD simulations using the GROMACS code (2019 version or higher).

## Author contributions

FJ, SIH, ZEH and NSG: conceptualization, methodology, performing analyses, interpreting results and writing – original draft. SIH, JS, SCS, YTG, ZEH and NSG: supervision, writing – review, and editing. All authors read and approved the final manuscript.

## Conflicts of interest

There are no conflicts to declare.

## Supplementary Material

RA-012-D2RA01892F-s001
