# Supplementary material for: Molecular basis of transport of surface functionalised gold nanoparticles to pulmonary surfactant
Source: RSC Adv. 2022 Jun 17;12(28):18012–21. doi: 10.1039/d2ra01892f (PMC9205331; doi:10.1039/d2ra01892f)
Supplement: RA-012-D2RA01892F-s001 [file RA-012-D2RA01892F-s001.pdf]

Electronic Supplementary Material (ESI) for RSC Advances.
This journal is © The Royal Society of Chemistry 2022

Electronic Supplementary Information: Molecular Basis of Transport of Surface Functionalised Gold Nanoparticles to Pulmonary Surfactant

Fengxuan Jiao[1£], Sheikh I. Hossain[2£], Jianbing Sang[1], Suvash C. Saha[3], YuanTong Gu[4], Zak E Hughes[5*], Neha S Gandhi[6*]

[1]School of Mechanical Engineering, Hebei University of Technology, Tianjin 300401, PR China

[2]School of Life Science, University of Technology Sydney, 81 Broadway, Ultimo, NSW 2007, Australia

[3]School of Mechanical and Mechatronic Engineering, University of Technology Sydney, 81 Broadway, Ultimo, NSW 2007, Australia

[4]School of Mechanical Medical & Process Engineering, Queensland University of Technology, 2 George Street, GPO Box 2434, Brisbane, QLD 4000, Australia

[5]School of Chemistry and Biosciences, The University of Bradford, Bradford, BD7 1DP, UK

[6]Centre for Genomics and Personalised Health, School of Chemistry and Physics, Queensland University of Technology, 2 George Street, GPO Box 2434, Brisbane, QLD 4000, Australia

£ authors with equal contribution

*Corresponding authors: Zak Hughes <Z.Hughes@bradford.ac.uk>; Neha S. Gandhi neha.gandhi@qut.edu.au

Extended Simulations

In order to ensure that the simulations had equilibrated by ~750 ns some systems were simulated for an extended to 3000 ns. Figure S1 shows the position of the Au nanoparticles (NPs) as a function of simulation time. After a short equilibration period (<100 ns) the AuNPs maintain a constant position over the full 3000 ns. The surface tension (as an average over 75 ns time periods) as a function of simulation time is shown in Figure S2. While the surface tensions fluctuate an equilibrated values is typically reached by <500 ns. Overall these extended simulation results confirm that 1050 ns is a suitable timescale for the determination of parameters considered in this work.

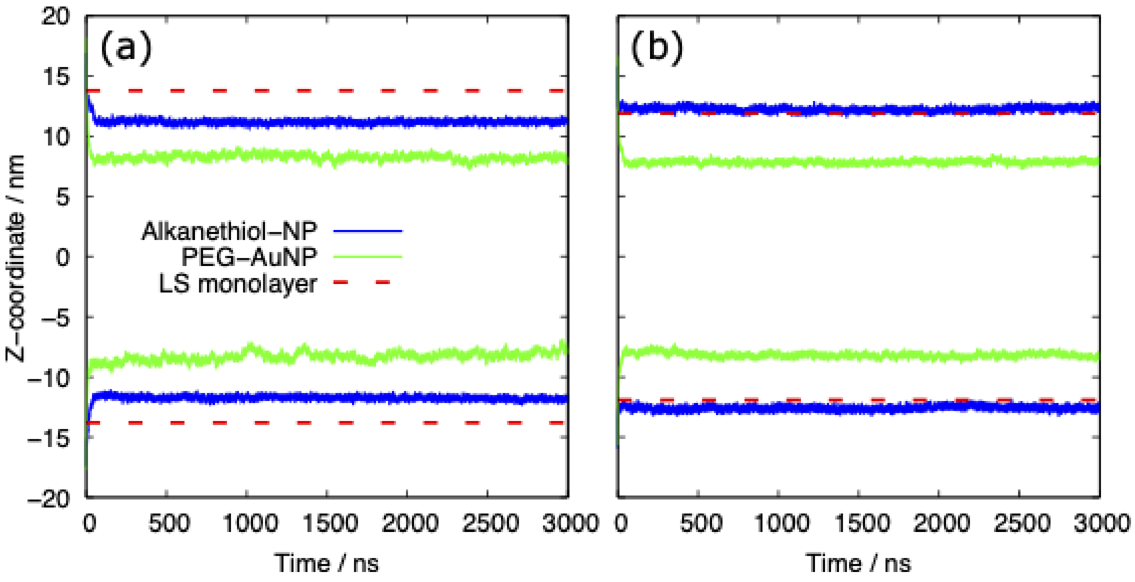

Fig. S1: The z-coordinate of the centre of mass of the AuNP as a function of simulation time for (a) the compressed (APL=0.48 nm^2) and (b) expanded (APL=0.54 nm^2) phases. The centre of mass of the lipids in each monolayer is shown by the dashed red lines. Representative data is shown for systems with a chain density of 2.33 chain nm^{-2} and a chain length of 2.

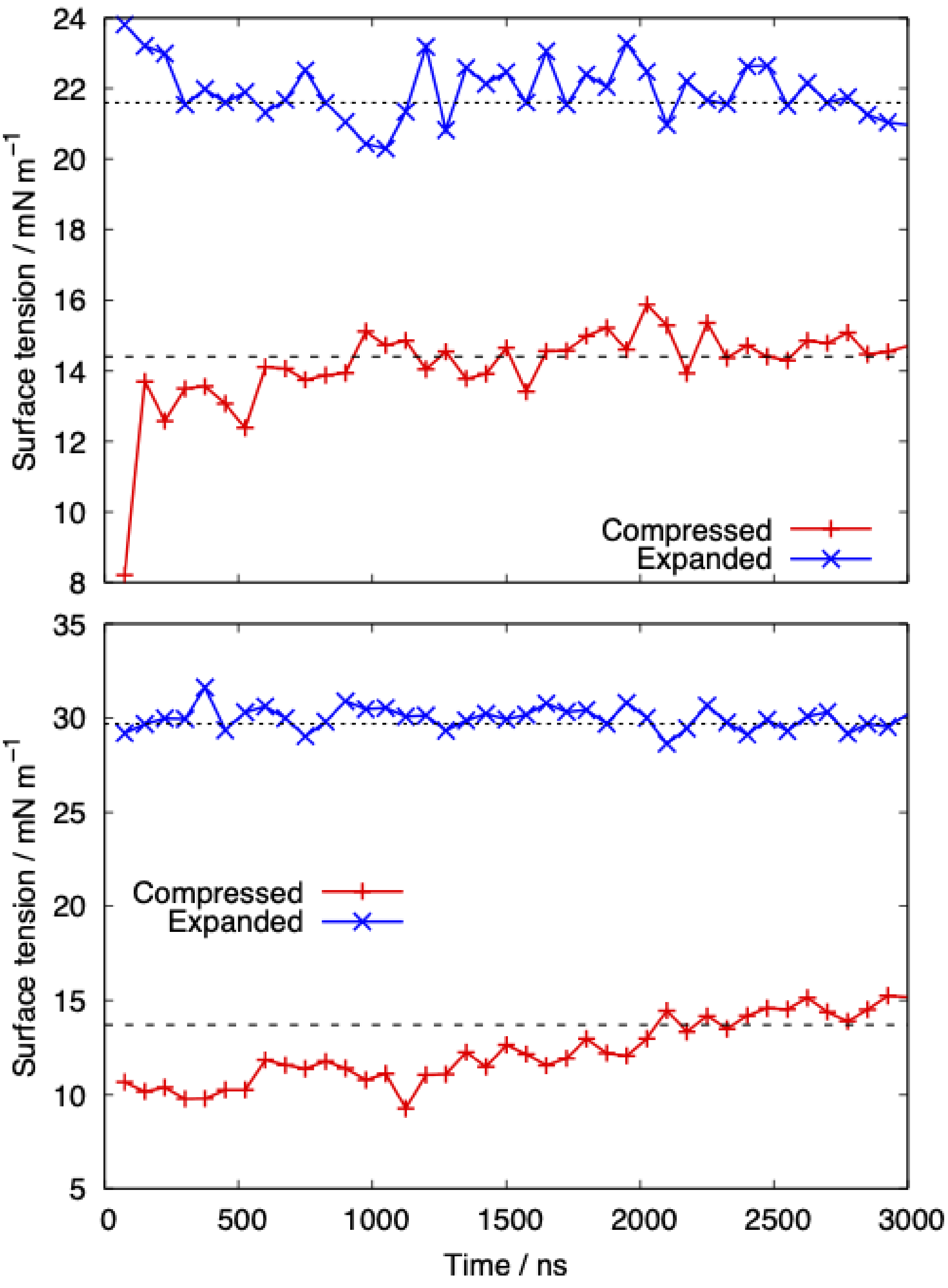

Fig. S2: Average of the surface tension as function of time for the extended simulations: (a) the alkanethiol AuNP systems and (b) the PEG-AuNP systems. Representative data shown for systems with a chain density of 2.33 chain nm^{-2} and a chain length of 2. The dashed lines show the surface tension for each system determined from the average over all runs.
